# Midwives’ perceptions of being ‘with woman’: a phenomenological study

**DOI:** 10.1186/s12884-019-2548-4

**Published:** 2019-10-21

**Authors:** Zoe Bradfield, Yvonne Hauck, Ravani Duggan, Michelle Kelly

**Affiliations:** 1 School of Nursing, Midwifery and Paramedicine, Curtin University, GPO Box U1987, Bentley, 6845 Western Australia; 2 Department of Nursing and Midwifery Education and Research, King Edward Memorial Hospital, Subiaco, Western Australia

**Keywords:** ‘With woman’, Midwifery, Philosophy, Phenomenology, Professional identity

## Abstract

**Background:**

Being ‘with woman’ is a central construct of the midwifery profession however, minimal research has been undertaken to explore the phenomenon from the perspective of midwives. The aim of this study was to describe Western Australian midwives’ perceptions of the phenomenon of being ‘with woman’ during the intrapartum period.

**Methods:**

Descriptive phenomenology was selected as the methodology for this study. Thirty one midwives working across a variety of care models participated in individual interviews. Giorgi’s four stage phenomenological approach was employed to analyse data.

**Results:**

Three themes were extracted 1) Essential to professional identity; 2) Partnership with women; and 3) Woman-Centred Practice. Midwives described the importance of being ‘with woman’ to the work and identification of midwifery practice. Developing a connection with the woman and providing woman-centred care inclusive of the woman’s support people was highlighted.

**Conclusions:**

For the first time, we are able to offer evidence of how midwives understand and perceive the phenomenon of being ‘with woman’ which has theoretical and practical utility. Findings from this study provide evidence that supports expert commentary and confirms that midwives conceptualise the phenomenon of being ‘with woman’ as essential to the identity and practice of the profession. Some previously identified ‘good midwifery practices’ were revealed as practical manifestations of the phenomenon. This new knowledge facilitates clarity and provides evidence to support statements of professional identity, which is useful for the development of educational curricula as well as supporting graduate and professional midwives. The findings emphasise the importance of the development of language around this important philosophical construct which permeates midwifery practice, enhances professional agency and supports the continued emphasis of being ‘with woman’ with new understanding of its applied practices in a variety of care models.

## Background

Being ‘with woman’ (WW) is a central tenet of midwifery philosophy and practice. Statements from peak midwifery professional bodies around the world reference the importance of working in partnership with women and providing care that is woman-centred [1–5]. The Australian College of Midwives’ professional philosophy statement reads “Midwife means ‘with woman’: this underpins midwifery’s philosophy, work and relationships” [1]. This statement explicates the fundamental importance of being WW to the profession of midwifery. Australian professional practice standards highlight the importance of being WW to underpin the practice of midwifery [6–8] and the International Confederation of Midwives also confirms the importance of being WW by working in partnership with women [9].

Despite the significance of being WW to the profession of midwifery there has been little research that has specifically focussed on the constructs or practice of being WW. One midwife academic from the United States (US), published a literature review as a precursor to her doctoral research that referenced studies exploring women’s perceptions of ‘good’ midwifery care [10]. From the research describing women’s perceptions and experiences of midwifery care, Hunter developed a set of characteristics that she asserts were attributable to being WW such as: “knowledge and professional expertise, sensitivity, personal attention, nurturance, support and guidance, advice and information, and; a trusting guide” [10]. Hunter presented a hermeneutic phenomenological study of birth poetry written by ten midwives which revealed that being WW is a specific and unique component of midwifery care [11]. Later, Hunter (2009) conducted a descriptive correlational study that surveyed 238 low risk women who gave birth in a hospital or birth centre in the US and asked them to rate their experience of being ‘with’ their midwife using Lehrman’s Positive Presence Index Scale. With the contention that therapeutic presence was an essential component of being WW, results confirmed that women who began or proceeded to birth in a birth centre environment rated higher presence scores than their standard hospital labour ward counterparts [12].

A recent integrative review confirmed that the midwife-woman relationship characterised by inclusiveness, sensitivity and care is an important element of being WW [13]. Australian research exploring the intersection of being WW in the context of the private obstetric model recently revealed that in that clinical context, the unique triad of relationships between the woman, midwife and obstetrician was an important feature that could facilitate and challenge midwives being WW [14].

Further research, not specifically aimed to explore the concept of being WW; but midwifery practice in general, serendipitously discovered constructs of being WW. Davis and Walker (2011) interviewed 48 case-loading New Zealand midwives to explore how these midwives constructed their care. The authors concluded that in being WW, midwives were able to provide woman-centred care that enabled women to move through a variety of maternity contexts and settings according to their individual needs [15]. Furthermore, Barker’s study (2010) reported on the analysis of interviews conducted with seven midwives from the United Kingdom (UK) that explored experiences of providing emotional support to women becoming mothers. Findings revealed that midwives face a dilemma between being ‘with institution’ and fulfilling the needs of the ‘system’ that manages healthcare services versus being WW [16].

Midwifery leaders from the UK, US and Australia have emphasised the importance of being WW to midwifery practice citing the historical context of WW practices. Authors heralded the professionalisation of midwifery in turn-of-the-century publications maintaining that being ‘with woman’ was an appropriate cornerstone for the development of professional philosophy [17–19]. In recent times, being WW is offered as the antidote to the techno-rational medical model that problematizes women treating childbearing like a ‘condition that requires curing’; and instead facilitates a woman-centred approach to enhance agency in women and midwives alike [20].

The woman-midwife relationship has been asserted as a significant construct of being ‘with woman’ and is a widely reported feature in professional and editorial commentary [21–25] This knowledge provided by midwifery leaders provides the impetus to deliver an evidence-informed understanding of how and in what ways, the woman- midwife relationship forms part of being WW in the context of the various maternity settings that midwives work.

Despite the centrality of being WW to the thinking, theory, and practice of midwifery, a recent integrative review revealed there has been no research to date that has explicitly sought to explore or qualify this phenomenon from the perspective of midwives [13]. Having an understanding of the constituents of the phenomenon of being WW from the perspective of midwives would offer a unique and important conceptualisation of this foundational professional philosophy. Considering the asserted centrality of the woman- midwife connection to the philosophy of being WW, as well as the identified gaps in knowledge, it was considered prudent to explore the understanding held by midwives working in a variety of settings. Consequently, the aim of this study was to explore midwives’ perceptions revealed through experiences of the phenomenon of being WW in the intrapartum period. This research forms one component of a series of studies which; as well as describing midwives’ perceptions of the phenomenon of being WW here, also included distinct components that sought to understand midwives’ experiences of being WW in the context of the various models of maternity care within Western Australia (WA). Findings of the intersection of being WW in the context of the various models have been published elsewhere [14, 26, 27].

## Methods

This study used a descriptive phenomenological design to explore midwives’ perceptions and experiences of being WW during labour and birth. Developed by Husserl, phenomenology has its genesis in the discipline of philosophy. The adoption of the ‘phenomenological attitude’ unique to descriptive phenomenology, supports thoughtful and methodological exploration; and is useful to explore the ‘way things appear’ in relation to the way phenomena are experienced [28]. Central to descriptive phenomenology is the adoption of a phenomenological attitude which is characterised by the researcher’s openness toward the phenomenon under study facilitated through a suspension of the researcher’s natural attitude, perceptions or experiences. In contrast, the methodology of interpretive phenomenology, developed from the phenomenological philosophy of Heidegger and Gadamer focuses on exploring the meaning of phenomena which is revealed by embracing the researcher’s experiences and perceptions of the phenomenon under study rather than suspending these [29–32].

It is widely asserted that descriptive phenomenology is useful to elicit rich descriptions from participants and reveals constituents of the same phenomenon as it is experienced by different individuals [28, 30, 33–35]. This stance was considered the ideal methodological approach given that little research has been conducted to specifically understand the phenomenon of being WW. Descriptive phenomenology is known to elucidate poorly understood aspects of phenomena from the perspective of the participants with lived experience, to share the distinct or essential features, which facilitates a general conception of the phenomenon [36, 37]. It is asserted that the methodology is useful for revealing essences of a phenomenon in a way that “...neither adds nor subtracts from the invariant intentional object arrived at, but describes it precisely as it presents itself” [38]. Because of the sparse nature of any previous empirical research on this phenomenon, it was considered important to keep as close to the midwives’ descriptions as possible which also guided the selection of this methodology. Amadeus Giorgi, a devotee of Husserl developed a data analysis framework to structure the phases of phenomenological research, adding rigor and transparency; his approach is utilised in this research [28]. This study was approved by Curtin University Human Research Ethics Committee (HREC) (approval number HREC 2016–0450).

### Participants

Midwives working in WA who had provided labour and birth care to women in any setting within the previous 12 months were recruited. The research was set in WA which has a two-tiered system of public and private care; within these systems there are three main models of midwifery care, where care is provided by an unknown midwife (UM), an unknown midwife under the direction of a known obstetrician (UMKO) and finally; a known midwife (KM). These models are further explicated in Table 1.

**Table 1:** Models of midwifery labour and birth care in Western Australia

	Unknown Midwife (UM)	Unknown Midwife Known Obstetrician (UMKO)	Known Midwife (KM)		
	Public Obstetric-led Midwifery Care	Private Obstetric-led Midwifery Care	Public Midwifery Group Practice (MGP)	Public Community Midwifery Program (CMP)	Privately Practicing Midwife (PPM)
	Woman attends hospital where labour care is usually^a^ provided by an unknown midwife	Woman attends hospital where labour care is provided by an unknown midwife managed by the woman’s known obstetrician	Woman contacts her known midwife and prepares for birth in planned place	Woman contacts her known midwife and prepares for birth in planned place	Woman contacts her known midwife and prepares for birth in planned place
Woman’s Planned Place of Birth					
Public Hospital	X	X	X	X	X
Private Hospital		X			
Public Birth Centre			X	X	
Planned Home Birth			X	X	X
Freestanding Birth Centre			X	X	X

^a^In some smaller rural or secondary public hospitals, it is possible that the midwife may provide antenatal care and serendipitously provide labour and birth care and so there may be opportunity for relationship, it is estimated that this would account for < 1% women in this model

Summarised data from WA Department of Health ‘Having a Baby’ [39]

The study was initially advertised at a local midwifery conference with over 170 midwives in attendance. As is the recommended practice in phenomenological research, participants were purposively sampled to ensure recruitment of midwives with a lived experience of the phenomenon under study [28, 40]. Additionally, an effective strategy to connect the researcher to further participants who had a lived experience of the phenomenon working in a variety of settings was through the use of snowball sampling [41] . Prior to commencing interviews, prospective participants were emailed an information letter and written, informed consent was obtained by the primary researcher (ZB). A comprehensive demographic profile is presented in Table 2 below. In total, 31 female midwives participated; they had between 3 and 35 years of midwifery experience and their ages ranged from 35 to 62 years. Ten midwives worked in midwifery continuity models where labour and birth care is provided by a ‘Known Midwife’; 11 were from private obstetric-led models where care is provided by an ‘Unknown Midwife and Known Obstetrician’; and a further ten worked in standard public models where care is provided by midwives not previously known by the women, or, ‘Unknown Midwives’.

**Table 2:** Participant demographic profile (*N* = 31)

Demographic variables	Participant numbers
Gender	
Female	31
Age	
30 to 40	9
41 to 50	9
51 to 60	12
61 to 70	1
Years of experience as a midwife	
< 5 years	3
5 to 10 years	7
11 to 15	6
16 to 20	1
21 to 25	3
26 to 30	7
31 to 35	4
Level of midwifery education	
Hospital–based diploma	12
Undergraduate midwifery degree	6
Postgraduate midwifery qualification	13
Other countries practiced midwifery aside from Australia	
England	7
Scotland	1
New Zealand	1
Current midwifery model	
Known Midwife (KM)	
Midwifery continuity models, 5 different metropolitan services (including private practice)	8
Midwifery continuity models, 2 different rural sites	2
Unknown Midwife Known Obstetrician (UMKO)	
Three different metropolitan private hospitals	11
Unknown Midwife (UM)	
Standard public care, 5 different metropolitan hospitals	8
Standard public care, 2 different rural sites	2
Previous Experience in Alternate Model?	
Yes	22
No	9
TOTAL	31

### Data collection

In depth, one-to-one interviews lasting between 45 and 90 min were conducted at a location and time convenient to the participants. Four midwives were interviewed over the telephone as they were based in rural areas; all interviews were digitally recorded and transcribed verbatim. Data were collected concurrently from midwives working in all models. Adopting a phenomenological attitude provoked a deliberate naiveté which enabled the researcher to follow the accounts of the participants; this provoked subsequent prompts or questions from the interviewer during the interview to further clarify and elucidate the descriptions offered. Participants were asked to describe their perceptions and experiences of being WW. Establishing constructs through the descriptions of hyletic characteristics of a phenomenon is central to descriptive phenomenology [42]. The opening question was “Please would you describe your perception of what it means to be with woman?” Midwives initially offered a sentence answer and then immediately proceeded to explain that being WW was ‘difficult to describe’. Interviewer prompts were offered and the strategy that elicited the rich descriptions was the following “If I (researcher) was a fly on the wall and I was to see you being WW what would I notice or see/ what would I not see; from that, can you describe your experiences of being WW?” It was then that midwives were able to describe their experiences of being WW, expounding what being WW ‘does’ or ‘looks like’; describing the phenomenon from all ‘angles’ including what it was and what it wasn’t.

The interviewer, currently a midwife academic, was known to two participants as past professional acquaintances. The interviewer had worked clinically with two of the midwives for only a brief period over 5 years earlier; the potential for influence on participant responses therefore was considered minimal. The process of bracketing is central to phenomenological research and involved suspension of any prior conceptualisations or personal assumptions about being WW; which facilitated immersion in the participants’ ‘lifeworld’ through their descriptions of their lived experience of being WW [43]. The interviewer was mindful to adopt a neutral body posture and tone of voice to avoid influencing participant responses [28, 36]. Additional strategies to enhance phenomenological reduction were undertaken, these included: writing field notes; recording of the interviewer’s philosophy and thoughts about being WW prior to commencement of data collection; maintenance of a research journal to reduce any pre-existing thoughts and to facilitate reflection after interviewing; and having regular meetings with the other members of the research team [44]. There is no pre-determined method for predicting the ‘required’ sample size within qualitative studies. Within phenomenological research there is differing assertions with suggestions of between 3 and 10 participants [45, 46], up to 20 participants [40] and between 3 and 30 participants [47] . Giorgi [38] maintains that the sample size is of less importance than the richness of description; and that there needs to be sufficient participants to identify a range of variation. We anticipated a larger sample size as we invited midwives working with labouring women in different models of care. A cautious approach was taken with regard to recruitment, to ensure that any differences described from participants working in the various models would be captured [48]. Data saturation was confirmed when no new concepts were revealed and was beginning to become evident after the 27th interview. A further 4 interviews were conducted to confirm no new concepts would be added [49] and to facilitate inclusion of midwives who had expressed an interest in participating in the study to share their experiences [41].

### Data analysis

Data analysis was scaffolded by Giorgi’s four stage phenomenological approach and although presented in a linear manner here, was in reality an iterative process. The first stage was data immersion; digital recordings of the interviews allowed the primary researcher to be ‘fully present’ and follow the lead of the participants’ descriptions during the interviews. Another strategy employed that facilitated data immersion was re-reading the interview transcripts and listening to the audio recordings. The second stage involved dividing data into concepts which involved the extraction of individual meaning units or conceptualisations. To facilitate the management and grouping of the large amounts of qualitative data, N-Vivo (v.11) data analysis software was used. In the third stage, the focus was the organisation and transformation of the data which facilitated the arrangement and expression of the data into commonalities of participant descriptions, revealing the necessary and invariant features of the phenomenon. In the fourth stage, an expression of the constituents of the phenomenon was achieved. In this final step, use of the researchers’ ‘disciplinary intuition’ enabled statements to be transformed into concepts articulating the constituents of the phenomenon [28].

The constituents of the phenomenon were supported by participant quotations [28] which were italicised in text with a unique identification code indicating the participant number and model of care worked in (KM, UM, UMKO P 1–11): Known Midwife (KM), Unknown Midwife (UM) and Unknown Midwife/Known Obstetrician (UMKO). The themes and subthemes were represented across each of the models, which are supported by a selection of quotes that have been chosen to facilitate a succinct presentation of the findings. To enhance a concise display of participant descriptions, non-essential words were omitted and are indicated within the text by an ellipsis (…). Words that have been added to elucidate participant vernacular or provide conversational context are not italicised and indicated by square brackets [] [41]. The first author analysed all transcripts, in addition, at least one other member of the author team concurrently and independently analysed each transcript. Initial findings were conferred in a research team meeting and discussed until consensus was reached around themes and sub-themes which added rigor to the data analysis [41].

## Results

**Fig. 1:**
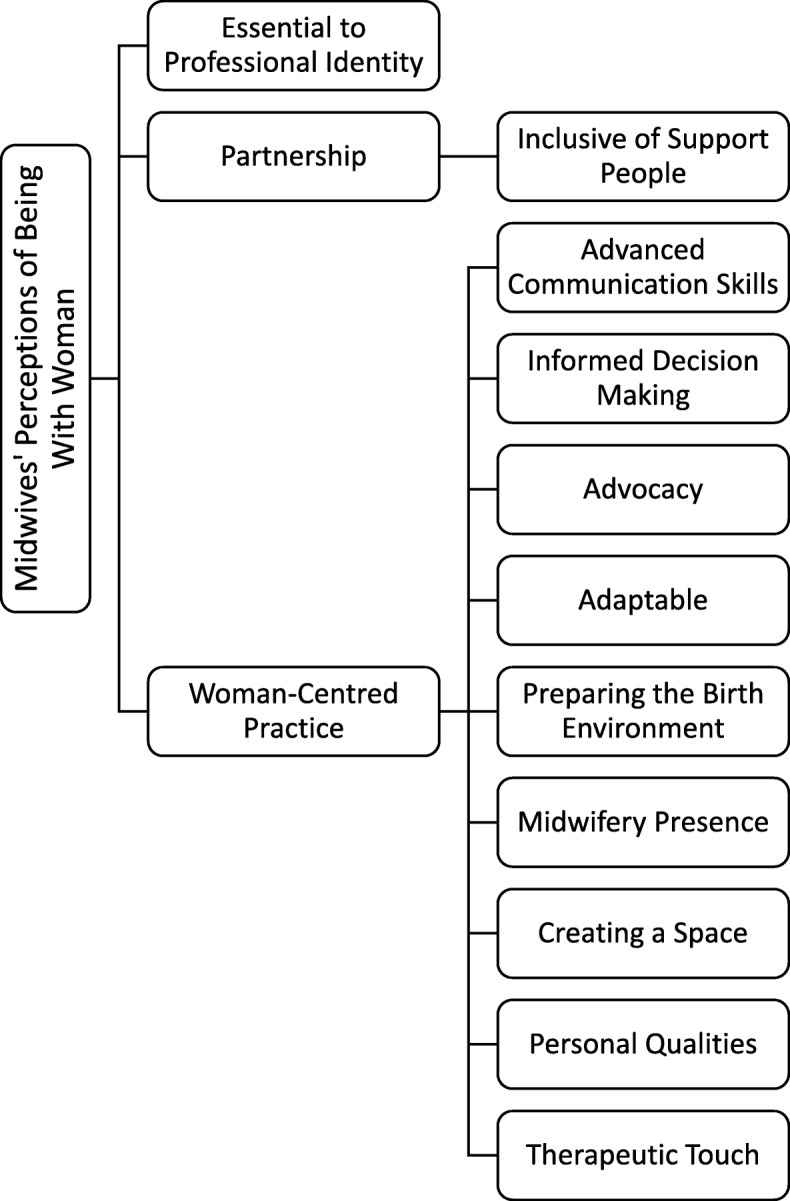
Themes and Subthemes - Midwives’ Perceptions of being ‘With Woman’

Through the in-depth interviews, midwives offered rich descriptions of the phenomenon of being WW that were supported with recounted experiences of being WW during labour and birth throughout their years of being a midwife. Three main themes were identified, 1) Essential to Professional Identity, 2) Partnership and 3) Woman- Centred Practice along with corresponding subthemes (Fig. 1).

Although midwives were asked to describe the phenomenon of being WW during the intrapartum period, midwives working in each of the models asserted that being WW was not restricted to the period of intrapartum care; rather, that being WW is an essential component of midwifery practice across the continuum: *…I absolutely do think that with woman is more than just an intrapartum* [phenomenon] *because then I support them to have a really good breastfeeding experience and a great start to being a mum and a great start to being parents* (KMP6).

Midwives repeatedly reflected on the challenge of describing the phenomenon of being WW: ... *it is hard, it* [is] *really interesting when I’ve been thinking, how do you describe it? It’s really difficult* (UMP2). One midwife offered some insights into the source of the challenge and referred to the practise-based nature of midwifery work: ‘*Cause you don’t actually put it into words,* [you] *put it into action* (KMP6). This reveals the embedded nature of this philosophical construct to the practices of many midwives.

### Essential to professional identity

Being WW was described as a necessary and integral feature of midwifery practice: *Yes it* [being WW] *is absolutely essential because without being ‘with woman’ you’re not a midwife, you’re just a person doing the job... that doesn’t actually provide true midwifery care* (KMP9). The descriptions provided by midwives confirmed that practice grounded in being WW is the identifying characteristic of midwifery care rather than the individual clinical tasks that midwives performed: *You couldn’t do your job without being with woman, you have to be with woman...* [it’s] *very, very important…* (UM P7). This stance was confirmed when midwives described experiences of working with midwives where tasks were performed without being WW: *It* [not being WW] *looks disconnected, it looks directed, she* [the midwife] *stands at a distance, she gives instruction from a distance. There’s not a lot of eye contact, there’s not a lot of engagement, there’s not a lot of sincerity or warmth in that relationship or room or in that moment you know* (KMP6). Another midwife reflected: *You can provide midwifery care and not be ‘with woman’, you can be ticking your lists and checking your room but not actually being ‘with woman’* (UMP6).

Midwives described that being WW was both fulfilling and satisfying: *It* [being WW] *is what I do it* [midwifery] *for, it’s what I go to work every day for*... (KMP6). Midwives were cognisant of the privilege of being WW: *I just love it* [being WW] ... *it’s a privileged job* [midwifery] *isn’t it? I just love being part of it and, and empowering them* [women] (UMKOP7).

### Partnership

Midwives confirmed the importance of developing a connection with the woman which was often described as developing rapport, a partnership or, a professional relationship. Although this was manifested in different forms within the diverse models represented, each reinforced the importance of connecting with the woman in order to be WW. In the KM model, where midwives were able to offer continuity of care across the continuum, the descriptions were*: I’m in partnership with her… the family, the woman and the midwife and it’s altogether* (KMP7). And from another midwife: *it* [being WW] *is built on a relationship of trust… Because you know them and because you’ve had time to develop the relationship and there’s trust there because they will let you in more*... *I think it’s just knowing... built on a relationship of trust* (KMP10).

In the remaining two models where midwives provided labour care for women not previously known to them, the emphasis on building a connection with the woman was equally as strong with a focus on being effective: *I put a lot of effort into you know building that rapport and building that trust* (UMKOP11). Effective rapport building resulted in partnership: *“... through your relationship with them you know that the woman trusts you, like a journey next to her, like a partnership [the woman] will listen to what you’re saying and taking it on board* (UMPKOP3).

One midwife offered strategies that she used to develop a quick connection with women she was caring for in labour:

*So you have a tool kit that you use to develop your relationship with* [women] *by introducing who you are, what you do and ... you start to develop that relationship. And being with them, being at their bedside, being attentive to them and to their partners … we are sharing ourselves with each other so then that builds a relationship and trust* (UMP7).

This was supported by another participant who described how she used the ‘tasks’ of midwifery in a way that facilitated building an initial rapport seeing it as an opportunity to convey respect and trust:

“... [although] *it’s wonderful if you have had previous introductions, it’s not necessary* [for being ‘with woman’]. *It* [meeting a woman for the first time] *is a good way to have the ‘excuse’ to be at her bedside as well, to be there actually engaging, doing some of those little menial things we do but that’s what you’re there at the bedside [for] and that’s the opportunity to get that rapport and opening going* (UMP9).

Midwives also reflected on the challenge of developing connection with women who present in established labour but expressed the importance of the partnership that facilitated trust*: … that’s hard, that building up that relationship. Again I think you’ve got to get her trust as quickly as you can … building up that trust and relationship really quickly but dealing with all the clinical stuff and showing that you know what you’re talking about and ... what you’re doing… showing a woman that you’ve got respect for her* (UMP5).

#### Inclusive of support people

In addition to building a partnership with the woman, midwives were emphatic that being WW meant providing care that was inclusive of the woman’s partner or support people: *So it’s just getting that one on one relationship with her and her partner that’s really important, and working together to get an agreed goal or outcome* (UMKOP10). Midwives shared strategies for encouraging partners to feel confident and important in the process of supporting the woman in labour: *Often you’ll find that* [partners] *step back. I always say to them you know ‘don’t move out my way, come on I can get around you. She’s number 1 in the room and you’re number 2…you need to be where you need to be’. The women like their partners to be supportive, so you know that’s kind of encouraging them to do what they need to do for their woman really* (KMP10). Midwives agreed that being respectful and inclusive of the woman’s partner or support people was important and further facilitated the partnership between the midwife and the woman: *… it is really important to include them … because they’re a very important person to that woman* (UMP4).

### Woman-centred practice

In an effort to explicate the experiences or ‘action’ of being WW, midwives described the features of being WW as a way of embodying the practice elements of the phenomenon which revealed actions that signified midwives were being WW. Midwives were clear that this required practising with a woman-centred approach which demonstrated their ability to tailor care around what the woman wanted: *one of the first things I do when I look after a woman is,* [ask] *what does she want from me?* (UMP10). This required midwives to acknowledge the competing demands and agendas that may impact on the woman from other sources such as hospital policies and preferences of other practitioners and align themselves with the woman’s agenda: *the woman is the centre and should remain the focus and the centre of your attention* (UMKOP1). Midwives in the continuity models described that providing care that is centred on what the woman wants is made easier by the strong relationship that has been forged:

*working in a continuity model it’s about knowing that woman what she wants in labour... being with woman I’ve had the opportunity to get to know her, to ask her the question and priorities for labour … so that when labour does happen, for me I don’t have to ask her questions at that time it’s more just about following her lead and knowing, already knowing what she wants to do and what her responses will be so there doesn’t have to be any questioning in labour* (KMP8).

Midwives working with women not previously known to them relied on their ability to build an effective partnership with the woman, and on birth plans which when supplied by the woman, helped midwives to quickly ascertain the woman’s priorities in order to tailor her care: *listening to what she wants for her baby, for her birth, labour, if she’s got a birth plan* (UMKOP6). In situations where a birth plan was not formalised, midwives moved to open dialogue about how care could be adapted to meet the woman’s needs:

*If she doesn’t have a birth plan I’ll talk about what it is that she wants because I’m there to facilitate whatever it is that she wants from her birth experience and the only way I know that is by asking her what she wants... ‘with woman’ for me means always going back to the source, going back to the woman and seeing what it is that she wants from me* (UMP10).

There were nine subthemes which constituted characteristics of providing woman-centred care to demonstrate the practice of being WW which will now be described.

#### Advanced communication skills

Possessing advanced communication skills facilitated midwives’ ability to adopt a woman-centred approach and to be WW. Participants described the breadth of skills such as listening, hearing, clarifying, talking, providing verbal encouragement and the effective use of silence. As one midwife described: *Lots of listening to her and her partner and what their expectations were* [which was] *to keep the room nice and calm with no one else in the room, we took the time to do everything that she wanted* (KMP5). Midwives reinforced that being open and listening to the woman further conveyed respect and developed trust between the woman and the midwife which facilitated partnership: *listening to her birth plan, her ideas, her fears, getting that trust and you can almost have like a silent communication, you can just meet her eye and give the ‘it’s ok’ and she relaxes* (UMKOP11). Midwives were able to provide insight into what an outsider might observe when they were practicing these skills: *So you would notice me looking at that woman, to see verbal and non-verbal responses, observing her, speaking slowly to her, softly to her, being sensitive to not trying to talk to her while she’s … very obviously busy with her mind* (UMP8).

As well as listening to and hearing what is important to the woman, midwives reflected that they were also required to communicate with women in ways that were appropriate to different circumstances as shared by this midwife: *So I do talk a lot… through a contraction when they’re challenged. I try to focus their* [women’s] *breath, their headspace and I find something about their strength or something about their breath or whatever I just continue to use that* (KMP6). Being WW included providing verbal encouragement: *supporting and reassuring them if they are doubting give them some tips along the way trying to make it inclusive* (UMP2). To be WW, midwives also used verbal encouragement to help women to reconcile unexpected outcomes during labour and birth: *encouraging them … that they’re not a failure which a lot of women for some reason think that they are* (UMKOP9).

Midwives agreed that being WW sometimes called for silence, a pause in the rhythm of labour care that respected the woman’s need for stillness to focus and re-centre herself: *Sometimes there’ll be no communication, that might be being ‘with woman’* [I avoid] *disturbing her labour, everything I offer should be helping her to achieve her goal* (KMP8). There was a sense of being comfortable with the silence and using intuition developed in the respectful partnership to ascertain what the woman needed and wanted in that moment: S*ometimes you can have silence for a long time but … it’s not an uncomfortable silence. Some women do go ‘into themselves’ and don’t want a lot of chatter around them or touching or anything like that, but again reading that body language… some people change in labour so this would be being ‘with woman’* (UMP5). Midwives emphasised the importance of holding the space and resisting the temptation to be ‘busy’, honouring the woman’s need for silence: *…even if the room’s dead silent we just sit there. We won’t … go anywhere or do anything else we’ll just sit there and be there, we don’t get kind of distracted with “oh well I might go and restock theatre if nothing much has happened”* (UMKOP10).

#### Informed decision making

Another way midwives were able to be woman-centred and demonstrate being WW was to spend time educating women to facilitate informed decision making: [the] *‘with woman’ thing is about them* [women] *having choice and them being the ones guiding what’s happening for them... facilitating their decision making* (KMP6). There was a sense that in many cases this required midwives to create an environment that encouraged women to become powerful and utilise the agency that is rightfully theirs: *it’s about empowering the woman, supporting them to make decisions* (UMP7). Creating an environment where women were able to be autonomous and aware of their agency, required midwives to adopt a supportive role: *just being there and letting her make the decisions but supporting her through that process as well is really important* (UMKOP8). It was acknowledged that women might make decisions about their care that were different to the midwives’ perceived ‘right choice’, which required a professional approach to respecting the woman’s choice: *they’ve made a choice to come to this model of care and sometimes it’s a choice that you don’t always agree with ... but it’s the woman’s choice* (UMKOP7).

#### Advocacy

Advocating for the woman was noted as an important manifestation of aligning with the woman’s agenda and being WW. By understanding each woman and what she wanted, midwives were able to facilitate care:

*... if something happens that goes off the path that she wanted and she hasn’t got the power to say it because she’s feeling vulnerable, she’s feeling frightened, she’s feeling embarrassed then it’s your place to actually advocate for her ... people coming in and wanting ruptured membranes or to start antibiotics when the woman actually doesn’t want antibiotics and she can’t say it herself because she’s too scared* (KMP9).

Midwives reflected that ‘stepping in the gap’ and providing advocacy was often met with resistance from obstetricians or at times, other midwives. Being willing to tolerate any push-back or as this midwife describes it as, ‘noise’, was an important part of aligning with the woman’s agenda: *...it’s all about being with woman, being an advocate....* [midwives have] *to put up with the noise or the huffing and the puffing* (UMP4). It was clear that midwives understood the confidence required to advocate for women and were often aware that women valued this advocacy: *you have to be the advocate and really speak up and* [be] *confident doing that.... because the woman sometimes says things to you afterwards and says thanks for speaking up for me* (UMKOP8).

#### Adaptable

Offering care that was woman-centred and focused on the woman’s individual needs required midwives to be adaptable. Being adaptable required self-awareness and was seen as a measure of strength and capacity: *I take pride in the fact that I can adjust and I can actually be what that woman wants me to be because I am reading her cues properly and appropriately and correctly and I can move between environments and I’m actually ok with that* (UMP8).

Midwives relayed how the ability to be adaptable develops with increasing experience and also allows midwives to adapt across a range of settings: *I adapt to the environment and to the women ... that is the beauty about being experienced is that you are adaptable* (UMKOP3). Midwives described the nuanced ways of ‘reading’ what a woman needed in the moment as well as determining what needs were being met by other support people: *based on where they’re at, how they’re coping, what they need and what they’re getting from the other people that are in the room... I go by their behaviours, I go by what feedback they’re giving me, visual feedback like how they’re behaving ... they don’t say much to you in labour, then I go by what I do, how they respond to that… which might be them softening into whatever I’m doing* (KMP8).

#### Preparing the birth environment

Midwives also reported that preparing the physical environment or place for labour and birth according to the woman’s needs was important to being WW:

*… so they* [women] *would turn the lights off, they would go in and out of the bathroom, they would have music on... walking around and changing the furniture and leaning on stuff and you worked to what they did and if they weren’t sure you could give them suggestions* [so] *it felt like their room* (UMKOP5).

Another midwife describes what an outsider might observe: *you would* [notice] *silence*... *not many lights, their music. Even if we are in labour in birth suite, they are silent, on a mattress, I push the bed on the side, put the mattress on the floor. Mattress, bean bag, shower* (KMP7). Others described strategies to support privacy and normal birthing as a manifestation of being WW*:*

*…trying to get them to find their own place in that horrible, stark, bright, noisy, non-natural birthing environment. So the bathroom’s a good place I try and make that environment as conducive to normal birth as possible. So dimming lights, having quiet conversations and just rubbing her back or holding her hand or just sitting with my hand on her belly kind of timing contractions and listening to her voice, how she’s coping with them* (UMP3).

#### Midwifery presence

Midwifery presence was asserted as a fundamental way of being WW that focused solely on the woman and was offered in two parts, the first meant not leaving women to labour alone: *most of the time* [I am] *sitting by the woman, wherever she is I would be by her* (UMP7). There was agreement that the act of being physically present and focussed on the woman facilitated clinical assessment and care of the woman: ...*listen to the auscultation, have hands on palpation, rather than sit back and screen-watch... unless you’re with them and can get the whole clinical picture then you can’t really tell where they’re at progress-wise* (UMKOP4). In being WW, midwives suggested their clinical care was improved: ...*being present I think you get a better clinical assessment because you are with woman* (UMP4).

The second part of being present was not simply about being in the room: *it means being present with her but I don’t just mean present in the physical, but completely present* (KMP2). Midwives revealed that to an unaware observer, this might appear as though the midwife was ‘doing nothing’ and therefore the act of being present could be easy to miss and nuanced: *It’s so easy to miss it and not realise it if you was watching me because it’s done so quietly, it’s done so non-intrusive* (KMP1). Another midwife confirmed this and added that whilst at times, being WW did require ‘action’, midwifery presence was a valuable characteristic that often required a deliberate form of ‘inaction’: *well I can act if I need to but if I don’t need to act or do anything then this* [being present] *is the thing to be doing now* (UMP9). Being present also meant engaging with and being available by being attentive, aware, empathic and noticing subtle changes in the woman’s needs: *being present in the moment and making yourself available and open to whatever it is that she requires or needs to help her through* (UMP9).

#### Creating a space

In being present and WW, midwives created and prepared a space for the woman to be in. Different to making accommodations in the physical environment, preparing the birth space referred to creating an atmosphere free from unnecessary interruptions, that was safe and private: *allowing that space and private time, without everyone walking through – not like a production line* UMP2. This assertion was supported by another who confirmed that being WW required midwives to create and protect the birth space and described potential protagonists for a labouring woman: *It is ensuring that birth remains sacred for her and that really she comes to no harm just by the other course of events or by other people coming in … friends, family, staff, organisation needs of interfering with the process of birth* (KMP3). A consequence of holding and protecting the space of labour was described as facilitating the physiology of labour, a practice unique to midwifery *… allows her body to work so the whole thing [labour] can move forward because you’ve just made this space… it’s definitely unique to midwifery because there’s no other situation where you’re creating the space that will facilitate the outcome* (UMKOP9).

#### Personal qualities

Being WW required midwives to provide sensitive care which involved essential characteristics such as kindness, gentleness and respect: *part of being ‘with woman’… you care for women with kindness, with gentleness … with sensitivity, making sure that you treat those women as you want to be treated yourself. With respect, with dignity, gentleness. I’m pretty big on gentleness* (UMP8). Or as another midwife shared: *…simple things about being nice to people… it’s about actually being decent to people* (KMP3). Being kind demonstrated respect for women: *…respect is really important and they* [women] *will receive that respect really well and likely mirror that and you get trust from that person through that, through showing respect* (UMP4). Gentleness was also a way to demonstrate the respect inherent in being WW: *I’m just gentle with them* [women] *and appreciate them and respect them* (UMKOP8).

#### Therapeutic touch

A final manifestation of being WW shared by midwives in their support of women involved use of therapeutic touch sensitive to the woman’s needs: *being with woman you need to be able to touch her, to be able to calm her, massage, holding hands* (UMKOP6). Being self-aware and determining if the touch was therapeutic to the woman was essential:

*I use touch a lot. So I’m a reflexologist …* [I use] *aromatherapy oils, I like touch , I get involved with massaging their back or getting the partner involved in touch as well which I think most women like at certain times. I ask them and go through it with them to help them feel a bit more comfortable* (UMKOP8).

Midwives reflected on the impact of epidurals on the woman’s need for physical support and therapeutic touch: *if a woman is totally pain free she’s not giving that body language that she would if she didn’t have the epidural, there’s not that need to go and touch her and rub her back and stuff... perhaps we’ve lost a bit of ‘with woman’ in that way* (UMP5).

## Discussion

This study is the first to explicitly explore the phenomenon of being WW through in-depth interviews from the perspective of midwives providing intrapartum care in a range of maternity settings. Findings reveal the interconnectedness of this philosophical construct and the practice of midwifery. By using a phenomenological lens to explore the experiences of being WW, midwives offered rich descriptions of the attributes that characterise the phenomenon; as well as the way in which these are manifested through their practice of midwifery.

Our findings emphasised the importance of being WW to the identity and work of midwifery, such that midwives who were not displaying the characteristics and manifestations of the phenomenon were described as not ‘doing’ midwifery, or not ‘being’ midwives but merely persons providing care. The concept of midwives being ‘with woman’ rather than doing ‘to women’ has been previously explored where researchers have sought to identify the features of exemplary midwifery care [50]; describe midwives’ experiences of labour care [51, 52] or explore factors that are associated with women’s positive birth experiences [53, 54] which has led to serendipitous discoveries about being WW. Our study is the first of its kind which has sought to specifically and intentionally qualify being WW and offers insight into how midwives understand and experience this phenomenon. Our themes confirmed the interconnectedness of midwifery philosophy to midwifery practice and supports assertions within the professional philosophy statement published by the Australian College of Midwives [1]. Care that featured the characteristics of being WW identified the practice of midwifery and; in order to be doing ‘true midwifery’, participants asserted that midwives needed to be WW. This reflects how the two parts (midwifery philosophy and practice) are sections of the same whole. According to the participants in our study, to be ‘doing midwifery’ it is essential to be ‘with woman’.

Midwives in this study worked in different settings, had a range of qualifications and experiences from a variety of models and countries; however all conveyed the importance of being WW to their professional identity. Their rich descriptions reveal how the phenomenon is not unique to any particular model of care, length of experience or previous country of practice and is seen as an integral feature of midwifery practice. The findings of our research build upon an international study exploring the experiences of 48 New Zealand midwives working in a case-loading model, which revealed that being ‘with woman’ is a philosophical foundation that identifies the work and purpose of midwifery care regardless of the woman’s clinical risk or complexity [15]. The unique knowledge generated from this current WA study confirms that being WW is not dependent on place of birth or a specific model of care but is an identifying feature of midwifery care that occurs in multiple maternity settings.

Building a connection with the woman was expressed as a necessary requirement for being WW by WA midwives. How this connection was developed differed within the varying models of maternity care but the intention was the same; to build a trusting partnership with the woman that facilitated alignment between the woman and her midwife. The midwife-woman partnership is valued by midwives and is acknowledged as an important part of being WW which is confirmed in this study as well as in previous research [55–57] and professional commentary [58]. This study is the first to present an exploration of midwives’ understanding of the complexity, variety and role of developing a connection with the woman; considering the way this relates to the phenomenon of being WW in the context of three different models.

Previous research has focused on women’s experiences of the midwife-woman relationship, Australian research used a grounded theory approach to interview 14 women, the findings of which determined that women also valued the midwife-woman relationship, which influenced their decision to choose a model of care where this was more likely to be facilitated such as the known midwife models [59]. A metasynthesis of eight Swedish qualitative studies revealed the essential nature of the midwife-woman relationship that includes trust, support and mutuality which, authors asserted, maximise health outcomes for the woman and her baby [60]. Our research findings bring a complementary stance from the perspective of midwives and confirms that building a connection with women is an essential feature of being WW.

The participants in this WA study emphasised that to be WW, it was imperative for midwives to be woman-centred. This stance meant providing care that was focused on the woman, ensuring that it was individualised and fulfilled the needs of the woman rather than care providers or care institutions. This aligns with international midwifery leaders who argue for the importance of woman-centred care and suggests the intention is to bring the locus of control towards the woman rather than health professionals and institutions [22, 25, 61]. The mandate for woman-centred care is not a new concept in midwifery practice. Cross sectional research conducted in Japan with 482 women concluded that woman-centred care was valued by women and associated with higher rates of maternal satisfaction, perceptions of control and attachment to their newborns [62]. Being ‘with woman’ and providing woman-centred care are phrases frequently found in midwifery vernacular. Our findings illustrate from the perspective of midwives, how woman-centred care fits within the broader philosophy of midwifery and for the first time confirms that providing woman-centred care is an important construct of being WW.

A number of subthemes were revealed in the midwives’ experiences of woman-centred care which reflected being WW. The findings of this study have provided a collection of attributes that midwife participants asserted contribute to the provision of woman- centred care which was seen as the practical manifestations of being ‘with woman’. Each of these subthemes has been articulated in previous research expounding the characteristics of ‘good’ midwifery care but for the first time, has been identified as features of the applied practices of being ‘with woman’, collectively named by the midwives in our study, as woman-centred care.

All of the practical attributes of being ‘with woman’ identified by these WA midwives have been previously supported in research or professional commentary as ideal and desirable characteristics of midwifery care, some examples are shown here. Advanced communication skills that encompass listening, understanding, and speaking are recognised as essential skills to provide quality midwifery care [63, 64]. The role of the midwife in providing advocacy and facilitating the woman’s informed decision making has been presented in international studies emphasising the importance of their inclusion in midwifery care [65–68].

A modified Delphi Study conducted in 90 countries by the International Confederation of Midwives interviewed and surveyed midwifery educators and clinicians about necessary inclusions in midwifery curricula regarding knowledge, skills and professional behaviour. Findings confirmed an international consensus that midwifery programs should include education on important midwifery skills such as advocacy that enhances maternal empowerment, facilitation of informed decision making, the importance of the woman-midwife partnership, and the provision of woman-centred care [69]. Findings in this WA study revealed how being adaptable was regarded as a necessary attribute to provide care that was centred on the woman and an essential feature of being WW. A well-known UK study which conducted interviews with 11 midwives found that attributes such as being adaptable and self-aware contributed to professional resilience [70].

Being present and proximal to the woman during labour was captured in the WA midwives’ descriptions of being WW. Midwives also expressed the importance of providing therapeutic presence, described as a deliberate act of being engaged, attuned, respectful and observant. This theme is supported in a literature review [10] which included research exploring women’s experiences of birth and concluded that both the physical and emotional presence of the midwife during labour and birth formed part of being WW. Midwifery presence, designated as the embodiment of being WW is a key element designed to teach the important skills and attributes of midwives supporting women in labour [71]. Contrasting features such as being distant and disconnected were also highlighted by WA midwives in our study through descriptions of care that did not display the characteristics of being WW, where a midwife could be performing the ‘tasks’ of midwifery without being ‘present’ with the woman; highlighting the importance of being aware of the subtleties and nuanced nature of midwifery presence. This paradox was confirmed in a Swedish study that interviewed 18 women who had received midwifery care and shared stories where women had experienced similar task-oriented care describing the midwives as being ‘absently present’ [72].

Preparing and guarding a space for women to birth has been previously reported by midwifery leaders as a feature of midwifery care designed to create an environment where women can realise their capacity and agency [25, 73]. So too, the concept of therapeutic touch has been offered as an important strategy to demonstrate alliance and engage with the woman, to exhibit trustworthiness through gentleness, kindness and physical touch [74, 75].

It is evident that previous research and professional commentary has confirmed the importance of midwifery practices such as effective communication, advocacy, presence and partnership. A unique aspect and new discovery from our WA study is that rather than being isolated features of ‘good midwifery care’, these attributes are shown to also form part of the collective characteristics of woman-centred care which, it is asserted, is the practical manifestation of being WW. Our findings reveal midwives’ perceptions of how each of these important practices are nested within the broader conceptualisation of woman-centred midwifery practice, evidencing being WW and illustrating the embedded nature of professional philosophy within the work of midwifery.

In the modern techno-rational, task-driven landscape of health care, practices such as ‘presence’, which may be challenging to identify at first glance, risk being undervalued and eliminated by virtue of their inability to be quantified or measured. The findings from this research are timely in presenting a snapshot of contemporary midwifery care in a variety of settings and from the perspective of midwives who are being ‘with woman’ within these contexts. The message is clear that being WW is important both to midwives and to midwifery. For the first time, we have been able to offer a conceptualisation of the necessary and common components that midwives perceive are inherent to being WW. The synergies between midwives’ descriptions and the professional philosophy statement offered by the Australian College of Midwives are striking [1]. Midwives confirmed that being WW is an important and necessary feature of the profession (philosophical identifier); and not only describes the woman-centeredness of the work (midwifery practise) that we do but also the importance of with whom and how we do it (relationships).

The initial challenge that midwife participants in our study experienced in describing the phenomenon of being WW either directly, or through experience is important information. Despite the challenge, midwives were emphatic that being WW was essential to being a midwife and providing midwifery care. The juxtaposition of a phenomenon that is so central to midwifery and yet challenging to describe highlights the importance of these findings which for the first time, offer a substantial and applied understanding of the constructs of the WW phenomenon from the perspective of midwives themselves. It also draws attention to the importance of developing language around describing philosophical constructs that have applied relevance in our practice-based profession. The explicit descriptions provided in the findings of this study serve to articulate the manifestations or hallmark signs of being WW which are essential for the identification, preservation, and communication of a construct so important to midwifery. Equally, this unique and concrete knowledge serves as a basis for reviewing the development of the phenomenon of being WW into the future and acts as a basis for future research as the profession continues to develop and meet the needs of women and their families.

The findings will also be useful to advise midwifery curricula and inform the theoretical and skills-based, practical education of midwifery students. The new knowledge generated, articulates in a tangible way, the recognisable characteristics and manifestations of the phenomenon of being WW. This knowledge can be used to inform strategies to transfer the applied practices of being WW to students of midwifery programs which warrants further research.

### Strengths and limitations

A strength of this study is the inclusion of midwives who have worked in a variety of models, health services and who possess a range of qualifications and length of clinical experience. Despite the variety in these factors, the fact that the phenomenon was conceptualised similarly is noteworthy. Midwives self-selected into our study which may indicate that they hold distinctive views about being WW compared to others not interviewed, therefore findings must be considered within this context. The depth of our research discoveries from the experiences of these WA midwives, in conjunction with the rich description of the study context should enable readers to appraise the transferability of findings to varied maternity settings and across other countries.

## Conclusion

Findings from this study offer important knowledge about how midwives conceptualise the phenomenon of being with woman as the centre of their professional identity. Partnership between the woman and midwife is confirmed as an essential component of being with woman. Some previously recognised ‘good midwifery practices’ have been shown to contribute to midwife perceptions of woman-centred care which, it is revealed in this study, describe the practice attributes of being with woman. Knowledge generated in this study will be useful in the education sector and are also essential for the continuous professional development as the features of the phenomenon are linked in with the broader philosophy of midwifery. Leaders and clinical managers may benefit from insight into the embedded nature of midwifery philosophy into practice as findings confirm this importance and may inform future maternity service-design in a way that enhances midwives’ ability to work and be ‘with woman’.

## Data Availability

The data generated and analysed from the interviews conducted cannot be made publicly available due ethical concerns around the realistic potential of identifying individual participants from interview transcripts.

## Abbreviations

KM: Known Midwife Model
UK: United Kingdom
UM: Unknown Midwife Model
UMKO: Unknown Midwife Known Obstetrician Model
US: United States
WA: Western Australia
WW: With woman
